# Genome Analysis of 6222 Bacterial Isolates from Livestock and Food Environments in Spain to Decipher the Antibiotic Resistome

**DOI:** 10.3390/antibiotics14030281

**Published:** 2025-03-08

**Authors:** Marta Hernández, Álvaro Falcó-Prieto, Maria Ugarte-Ruiz, Pedro Miguela-Villoldo, Alain Ocampo-Sosa, David Abad, Marta Pérez-Sancho, Julio Álvarez, Rafael Dorighello Cadamuro, Mariana Alves Elois, Gislaine Fongaro, Alberto Quesada, Bruno González-Zorn, Lucas Domínguez, José M. Eiros, David Rodríguez-Lázaro

**Affiliations:** 1 Microbiology Department, Faculty of Medicine, University of Valladolid, 47005 Valladolid, Spain; alvarofalco@estudiantes.uva.es (Á.F.-P.); eiros@med.uva.es (J.M.E.); 2 VISAVET Health Surveillance Centre, Universidad Complutense de Madrid, 28040 Madrid, Spain; maria.ugarte@ucm.es (M.U.-R.); pedromig@ucm.es (P.M.-V.); maperezs@ucm.es (M.P.-S.); jalvarez@visavet.ucm.es (J.Á.); bgzorn@ucm.es (B.G.-Z.); lucasdo@visavet.ucm.es (L.D.); 3 Servicio de Microbiologia, Hospital Universitario Marqués de Valdecilla-Instituto de Investigación Sanitaria Valdecilla (IDIVAL), 39008 Santander, Spain; alain.ocampo@scsalud.es; 4 Centro de Investigación Biomédica en Red de Enfermedades Infecciosas (CIBERINFEC), Instituto de Salud Carlos III, 28029 Madrid, Spain; 5 Instituto Tecnológico Agrario de Castilla y León, Carretera de Burgos km 117, 47071 Valladolid, Spain; dabad87@gmail.com; 6 Microbiology Division, Faculty of Sciences, University of Burgos, 09001 Burgos, Spain; rdorighello@ubu.es (R.D.C.); malves@ubu.es (M.A.E.); 7 Research Centre for Emerging Pathogens and Global Health, University of Burgos, 09001 Burgos, Spain; 8 Laboratory of Applied Virology, Department of Microbiology, Immunology and Parasitology, Federal University of Santa Catarina, Florianópolis 88040-900, Brazil; gislaine.fongaro@ufsc.br; 9 Departamento de Bioquímica, Biología Molecular y Genética, Facultad de Veterinaria, Universidad de Extremadura, 10003 Cáceres, Spain; aquesada@unex.es; 10 Departamento de Sanidad Animal, Facultad de Veterinaria, Universidad Complutense de Madrid, 28040 Madrid, Spain

**Keywords:** antibiotic resistance, resistome, plasmid, one health, food safety, WGS

## Abstract

**Background/Objectives:** Antimicrobial resistance (AMR) poses a significant threat to global health and the economy, with projected costs ranging from $300 billion to $1 trillion annually and an estimated 10 million deaths per year by 2050. The food chain, from primary production to retail, represents a critical entry point for antimicrobial resistant bacteria into communities. This underscores the need for a coordinated “One Health” approach, integrating efforts in animal production, environmental health, and human healthcare to address this global concern. This study aimed to characterize the global resistome in Spanish primary production by sequencing 6222 bacterial genomes from animal origin. **Methods and Results:** Whole genome sequencing was performed on bacterial isolates collected from various farms and analyzed using a validated bioinformatic pipeline. The analysis revealed a diverse range of bacterial species, with *Enterobacteriaceae* being the most prevalent family. *Escherichia coli* was the most common species, followed by *Salmonella enterica* and *Pseudomonas aeruginosa*. This study identified 1072 antimicrobial resistance genes coding for 43 different classes of resistance, potentially conferring resistance to 81 antimicrobials. Additionally, 79 different plasmid types were detected, highlighting the potential for horizontal gene transfer. **Conclusions:** The resistome analysis revealed genes conferring resistance to various antibiotic classes, as well as antiseptics, disinfectants, and efflux pump-mediated resistance. This comprehensive characterization of AMR genes circulating in bacteria from primary production provides crucial insights into the ecology of AMR in Spanish livestock.

## 1. Introduction

Bacteria living in humans and animals are continuously exposed to antibiotics and can develop and transfer antimicrobial resistance (AMR). AMR is a major threat to human and animal health, increasing healthcare costs due to the longer duration of illness, additional diagnostic tests, the need for more expensive drugs, and increased mortality. It has been estimated that the economic cost of AMR to health care services will be approximately $300 billion to $1 trillion per year [1] and will kill 10 million people per year by 2050 [2]. The consequences of AMR in livestock have not been so precisely calculated, but can be estimated at an over 10% loss in the production of livestock by 2050 [3].

In addition to the inherent increase to the costs of production to the food chain, from primary production to the shelves of the supermarkets, represents a relevant gate for the entrance of AMR bacteria into the community. Therefore, efforts in animal production and environmental health must be coordinated with those established in human care to guarantee a holistic approach to control this global health concern. One of most promising alternatives is the implementation of a “One Health” strategy for the prevention and control of AMR dissemination. The term “One Medicine” was coined for the first time in 1796 by William Osler, a student of Virchow, and was taken up by Calvin Schwabe in 1984 in the third edition of his volume Veterinary Medicine and Human Health (a short introduction of the One Health History can be visit in https://www.cdc.gov/one-health/about/one-health-history.html, accessed on 3 December 2024). The concept grew to “One Health” in 2003 when the H5N1 avian influenza risk of a pandemic integrated the study of human and animal health. This holistic approach encourages the generation of knowledge of the biological elements necessary for understanding the evolution of AMR, including the microorganisms, the host organisms (humans or animals), and the environments involved [4].

With this scenario in mind, the World Health Organization (WHO) published the 23rd version of the Essential Medicines List (EML) in July 2023 [5] to provide guidance on antibiotic use for common human clinical infections and classify the antibiotics into Access, Watch, and Reserve (AWaRe) groups to reduce AMR. In addition, it has been currently encouraging the restrictive use of antibiotics in the agricultural sector and several antibiotic classes are forbidden for veterinary use. On the 13th of June 2023, the Council adopted a new recommendation aimed at stepping up European Union (EU) action to combat AMR in the fields of human health, animal health, and the environment. Regulation (EU) 2019/6 of the European Parliament and of the Council of 11 December 2018 on veterinary medicinal products lays down the rules for placing on the market, manufacturing, import, export, supply, distribution, pharmacovigilance, control, and use of veterinary medicinal products to ensure the proper administration and appropriate dosing of veterinary medicinal products to reduce AMR. A target for a 50% reduction of overall EU sales of antimicrobials for farmed animals and in aquaculture by 2030 has been included in the Farm to Fork Strategy [6].

Resistance to antibiotics in bacteria frequently appears soon after a new drug has been discovered due to misguided and continuous antibiotic pressure. AMR mechanisms by bacteria can be grouped into three strategies, including alteration of the antibiotic target site, modification or destruction of the antibiotic molecule, and inhibition of antibiotic binding to the target site. Moreover, the ability of bacteria to share genetic material facilitates the spread of AMR. Apart from the three general horizontal gene transfer methods, a recent newly described process allows *Staphylococcus aureus* pathogenicity islands (SaPIs) to parasitize phages and transfer themselves completely intact with bacterial DNA through termed lateral cotransduction [7]. Consequently, the characterization of the ecology and spread of AMR between bacteria is a key aspect to understand and control the transmission and dissemination of AMR. To date, there is not a wide study that considers the investigation of AMR in animal isolates, even though Spain was among the top 10 consumers of veterinary antimicrobials in 2017 (1.9% of the world totals). However, a reduction in sales of 41.83%, from 3028.62 to 1761.68 tonnes, was implemented in Spain between 2015 and 2017 [8]. In this study, we aim to provide relevant initial insight into the ecology of the AMR genes circulating in bacteria in primary production to characterize the global resistome in Spanish primary production by sequencing 6222 genomes of different bacterial species from animal origin and to discover the main flow of AMR genes in Spanish livestock.

## 2. Results

### 2.1. Bacterial Identification and Gene Distribution

**Figure 1:**
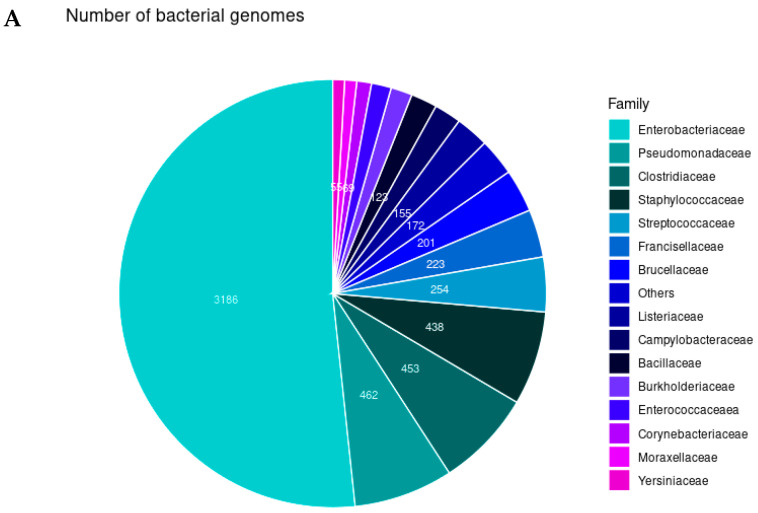

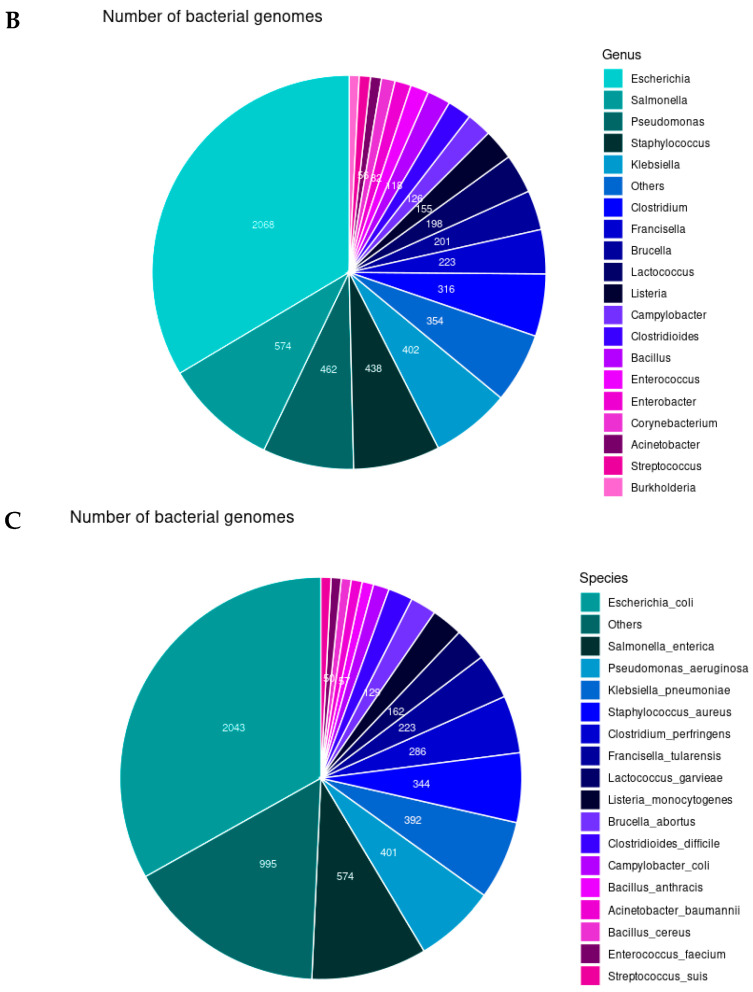
Main bacterial taxa used in this study: (**A**) Family, (**B**) Genera, and (**C**) Species sequenced in this study and the number of isolates.

After the WGS of the 6222 bacterial isolates, we identified 38 different bacterial families (51.33% *Enterobacteriaceae*, 7.43% *Pseudomonadaceae*, 7.30% *Staphylococcaceae*, and 7.28% *Clostridiaceae*), including 67 genera (33.63% *Escherichia*, 9.31% *Salmonella*, 7.49% *Pseudomonas*, and 7.36% *Staphylococcus*) and 176 different species (32.93% *Escherichia coli*, 9.22% *Salmonella enterica*, 6.44% *Pseudomonas aeruginosa*, 6.30% *Klebsiella pneumoniae*, and 5.79% *Staphylococcus aureus*) (Figure 1).

**Figure 2:**
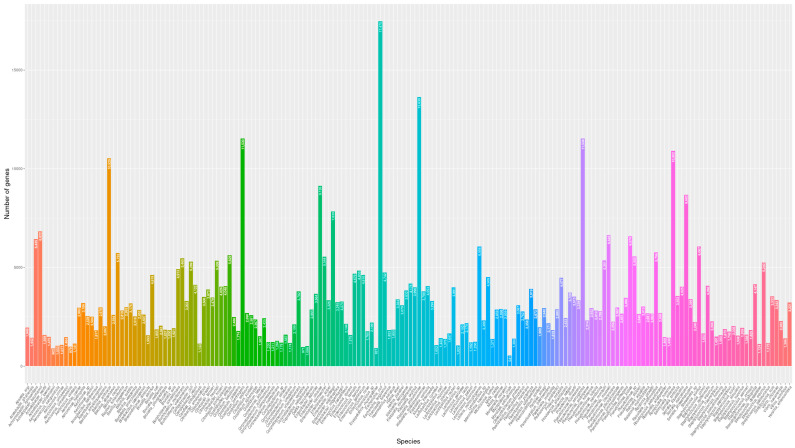
Total number of different genes identified in each bacterial species.

Once the isolate was identified from the draft genomes, we also annotated the genes that were assigned to a function by using Prokka [9]. A total of 577,904 different genes were recognized, mostly from *E. coli* (3.02% of the total genes), 2.36% of the genes were from *Klebsiella pneumoniae*, 2.00% from *Pseudomonas aeruginosa* and *Clostridium perfringens*, respectively, 1.89% from *Salmonella enterica*, 1.82% from *Bacillus anthracis*, and 1.5% from *Enterobacter cloacae* and other *Enterobacter* spp., *Serratia marcescens* and *Acinetobacter baumanii*. Other species harbor between 6000 and 541 genes; in the last case *Mycoplasma orale*. The total number of genes was calculated by bacterial species considering all the isolates of the same species (Figure 2).

There were no identified core genes (i.e., a gene that is present in all or nearly all individuals) with a threshold of 95%; however, some genes were found in a high prevalence in certain bacterial families, ranging from 70% to 85% in the case of *aph(3″)-Ib*, *aph(6)-Id*, *blaTEM*, *sul1*, *sul2*, *tet(A)*, and *tet(B)* for *Enterobacteriaceae*; as well as 70% for *tet(K)* in *Staphylococcaceae*; 80% for *tet(M)* in *Streptococcaceae*; and 75% for *aph(3″)-Ib*, *aph(6)-Id*, *tet(A)*, and *tet(E)* in *Aeromonadaceae*.

### 2.2. Plasmids

We have also been able to determine the presence of plasmid replicons in the characterized isolates that can be transferred between different species and transmit resistance to antibiotics. We identified 79 different plasmid types (200 counting the versions of each replicon): Col, FII, IncA, IncB, IncFIA, IncFIB, IncFIC, IncFII, IncHI1A, IncHI1B, IncHI2, IncHI2A, IncI-gamma, IncI1, Incl2, IncL/M, IncN_1, IncN3_1, IncP6_1, IncQ1_1, IncQ2_1, IncR_1, IncU_1, IncX1_1, IncX1_4, IncX2_1, IncX3_1, IncX4_1, IncX4_2, IncX5_2, IncY_1, p0111_1, pESA2_1, pSM22_1, rep1, rep10, rep11, rep13, rep14, rep15, rep16, rep18, rep19, rep2, rep20, rep21, rep22, rep24, rep25, rep26, rep28, rep29, rep3, rep32, rep33, rep38, rep39, rep4, rep5, rep6, rep7, rep9, repA, repUS1, repUS12, repUS15, repUS16, repUS18, repUS22, repUS27, repUS28, repUS33, repUS35, repUS4, repUS42, repUS43, repUS52, repUS54, and repUS70.

**Figure 3:**
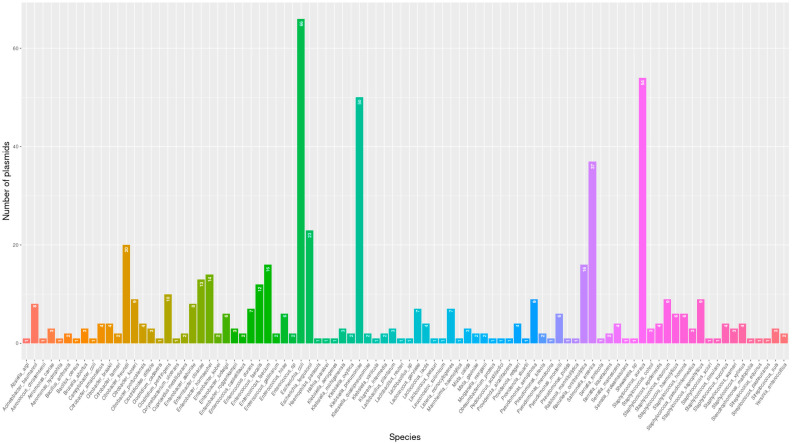
Number of plasmid replicons by bacterial species.

The most frequent distributed replicons were Col(MG828)_1, Col440I_1, repUS43_1_CDS12738(DOp1), IncFIB(K)_1_Kpn3, ColRNAI_1, IncHI2_1, ColpVC_1, and IncHI2A_1 in 28, 20, 20, 17, 11, and 10 different species, respectively. The replicons with more variants were Col, repUS43, and IncFIB. The bacterial families with a higher plasmid number included *Enterobacteriaceae*, *Staphylococcaceae*, *Enterococcaceaea*, *Streptococcaceae*, and *Clostridiaceae*, in which we identified 37, 22, 18, 12, and 10 different replicons, respectively. Members of *Acetobacteraceae*, *Alcaligenaceae*, *Caulobacteraceae*, *Comamonadaceae*, *Debaryomycetaceae*, *Flavobacteriaceae*, *Francisellaceae*, *Micrococcaceae*, *Mycoplasmataceae*, *Nocardiaceae*, *Paenibacillaceae*, and *Vibrionaceae* did not exhibit any plasmids. Furthermore, we have found a higher number of different replicons in the following species: *E. coli* (66), *St. aureus* (54), *K. pneumoniae* (50), and *S. enterica* (37) (Figure 3).

### 2.3. Resistome of the Animal Bacterial Isolates in Spain

**Figure 4:**
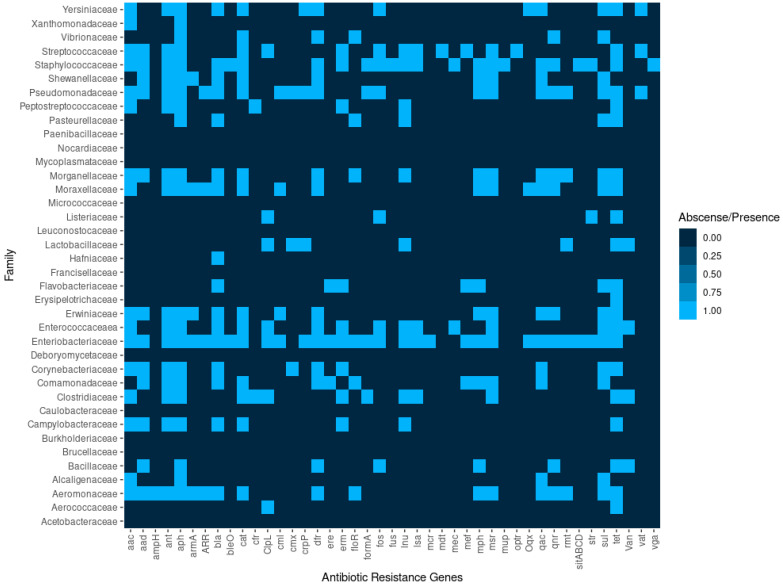
Types of AMR genes found in the different bacterial families in this study.

Our study describes for the first time the circulating resistome obtained by whole genome sequencing of 6222 animal-associated bacterial isolates from different origins in Spain. Overall, we identified 1072 genes associated with antimicrobial resistance coding 43 different classes of antimicrobial resistance, that can provide resistance to 81 antimicrobials (Figure 4). The AMR was clustered into 23 different classes considering the type of family of antibiotics (Figure 4).

**Figure 5:**
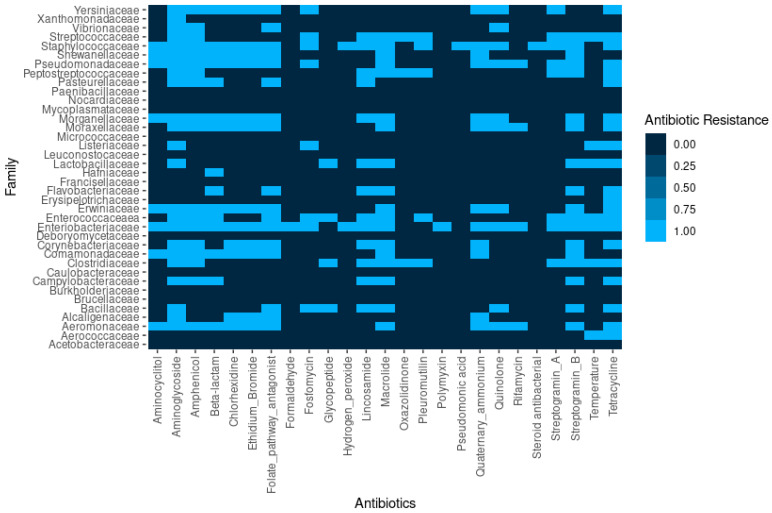
Types of antibiotics and disinfectants in the different bacterial families in this study.

In addition to AMR genes, genes coding resistance to antiseptics and disinfectants were also found in the genome, such as chlorhexidine, formaldehyde, hydrogen peroxide, pseudomonic acid, ammonium quaternary, and steroid antibacterial. We also identified the extrusion of antimicrobial compounds through efflux pumps that can be evaluated using ethidium bromide, considered a common substrate of efflux pumps in *Enterobacteriaceae* (Figure 5).

**Figure 6:**
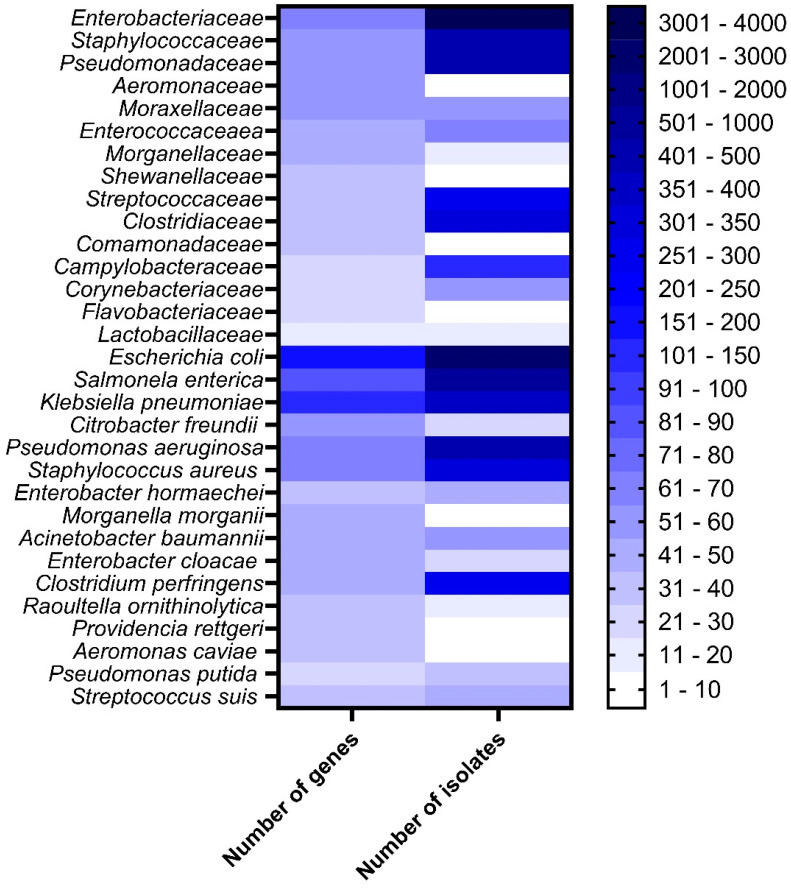
Bacterial taxa with the highest number of AMR genes identified in this study.

Interestingly, AMR genes were found in 27 (71.05%) and 117 (66.48%) of the bacterial families and genera identified in this study, respectively. This indicates that a high percentage of the bacterial taxa sequenced in this study harbors AMR genes. Figure 6 shows the bacterial taxa with a relevant number of resistance genes, whereas Table 1 shows the bacterial taxa without the presence of AMR genes. The five top antibiotic classes to which the isolates possess antimicrobial resistance genes (ARGs) were aminoglycosides (21.36%), beta-lactams (17.72%), tetracyclines (11.75%), cephalosporins (8.58%), and macrolides (5.32%); whereas only 3.26% of the bacterial species showed ARGs to carbapenems. Similarly, most of the genes were predicted to be resistant to tetracycline and doxycycline (4.76%), 3.17% to streptomycin, 2.89% to erythromycin, and 2.61% to ampicillin and amoxicillin.

These findings are concerning, as most of those species and the particular ARGs harbored are considered to be urgent threats under the recommendations of the U.S. Centers for Disease Control and Prevention (CDC) report [10].

Overall, 67.6% of the sequenced isolates could be considered to be multidrug resistant (MDR) bacteria, i.e., acquired non-susceptibility to at least one agent in three or more antimicrobial categories; while extensively drug-resistant (XDR) and pandrug-resistant (PDR) isolates were not found. The bacterial species with the major number of antibiotic resistance genes were *E. coli* (32.93% are MDR), followed by *S. enterica* (9.23%), *K. pneumoniae* (6,30%), *St. aureus* (5.79%), and *C. perfringens* (4.6%) (Table 2).

Similarly, the WHO also defined a list of pathogens belonging to the ESKAPE group to be treated as a priority (i.e., *Ent. faecium*, *St. aureus*, *K. pneumoniae*, *A. baumannii*, *P. aeruginosa*, and *Enterobacter* spp.). Of these, we found 49 strains of carbapenem-resistant *Acinetobacter baumanii*, 1114 cephalosporin-resistant *E. coli*, 269 cephalosporin-resistant *Klebsiella*, 26 vancomycin-resistant *Enterococcus faecium*, 186 isolates carbapenem-resistant *Ps. aeruginosa*, and 171 oxacillin-resistant *St. aureus*, respectively.

### 2.4. Bla Genes

**Figure 7:**
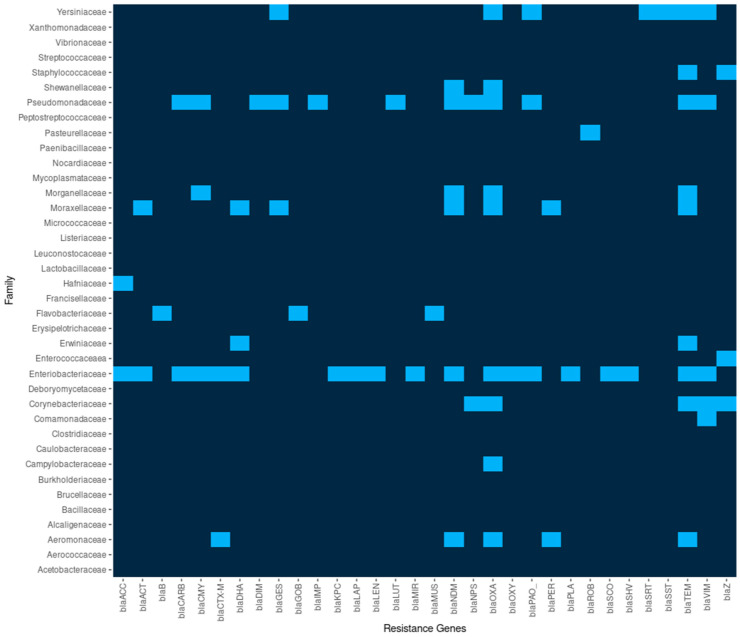
β-lactamases coding genes found in different bacterial families. The heat map indicates the absence or presence of a *bla* gene.

Resistance in Enterobacteriaceae producing extended spectrum beta-lactamases (ESBL), AmpC cephalosporinases, or carbapenemases are of major public health significance. Beta-lactamases impede the treatment with penicillins and derivatives that are mainly present in enterobacteria and pseudomonads and are encoded by *bla* genes. We found 32 different *bla* genes: *bla_ACC_*, *bla_ACT_*, *bla_B_*, *bla_CARB_*, *bla_CMY_*, *bla_CTX-M_*, *bla_DHA_*, *bla_DIM_*, *bla_GES_*, *bla_GOB_*, *bla_IMP_*, *bla_KPC_*, *bla_LAP_*, *bla_LEN_*, *bla_LUT_*, *bla_MIR_*, *bla_MUS_*, *bla_NDM_*, *blaNPS*, *bla_OXA_*, *bl_aOXY_*, *bla_PAO_*, *bla_PER_*, *bla_PLA_*, *bla_ROB_*, *bla_SCO_*, *bla_SHV_*, *bla_SRT_*, *bla_SST_*, *bla_TEM_*, *bla_VIM_*, and *bla_Z_*. The number of bacterial species with *bla_TEM_* were 27 (16.98%), bla_OXA_ 22 (13.84%), *bla_NDM_* 12 (7.55%), *bla_VIM_* 11 (6.92%), *bla_CMY_* 9 (5.66%), *bla_CTX-M_* 8 (5.03%), and *bla_Z_* 8 isolates (5.03%). Among β-lactamases with versatile hydrolytic capacities are the carbapenemases that produce bacteria resistant to carbapenems, a group of antibiotics used to treat serious infections. Carbapenemases can be grouped into Ambler class A (e.g., *K. pneumoniae* carbapenemase (KPC)), class B (e.g., Verona integron-encoded metallo-β-lactamase (VIM), New Delhi metallo-β-lactamase (NDM), and imipenemase (IMP)), and class D (e.g., oxacillinase-48 (OXA-48)). The presence of carbapenemase genes has been determined in different isolates by the presence of *bla_GIM_*, *bla_IMI_*, *bla_IMP_*, *bla_KPC_*, *bla_NDM_*, *bla_OXA-48-like_*, *bla_SIM_*, *bla_SPM_*, and *bla_VIM_*. We identified 2 *bla_IMP_*, 13 *bla_KPC_*, 45 *bla_NDM_*, 766 *bl_aOXA_*, and 66 *bla_VIM_*. In total, 892 carbapenemase-producing isolates were identified (Figure 7). *K. pneumoniae*, *E. coli*, *P. aeruginosa*, *A. baumannii*, *Serratia marcescens*, *Ent. cloacae*, *Ent. hormaechei*, and *S. enterica* were the species carrying the major number of carbapenemases.

### 2.5. MDR Correlation of Bacteria

In our study, 841 MDR bacterial isolates (45.67%) were identified, a significantly higher number and percentage than a similar study conducted in Portugal, and 70 MDR bacterial isolates (21.2%) from poultry and pig wastes [11], probably due to the higher quantity of samples analyzed and the period of time facilitated obtaining a wider range of bacterial AMR strains. Karczmarczyk et al. identified 100 MDR *E. coli* isolates on Irish cattle farms with resistance to aminoglycosides (100%), tetracycline (99%), sulfonamides (98%), beta-lactams (82%), and phenicols (9%) [12]. Our data encompasses a broader range of bacterial families, revealing resistance to aminoglycosides (21.36%), beta-lactams (17.72%), tetracyclines (11.75%), cephalosporins (8.58%), and macrolides (5.32%). Our sample collection includes a more diverse array of bacteria and environment sources, compared to only cattle, providing a more representative picture of MDR patterns. The most prevalent MDR bacteria containing these antibiotic classes were *E. coli* and *K. pneumoniae* (aminoglycosides, beta-lactams, and cephalosporins), and *E. coli* and *St. aureus* (tetracyclines and macrolides), being still *E. coli*, the bacterial species more frequently found.

## 3. Discussion

### 3.1. Microbial Community Structure

Different studies have shown different patterns of microbial community structure. Despite the metagenomic approach, the study developed by Liu et al. found five main families, including Moraxellaceae, Enterococcaceae, Enterobacteriaceae, Comamonadaceae, Pseudomonadaceae, and six main species, including *Ent. cecorum*, *Ent. faecium*, *Erysipelotrichaceae bacterium* MTC7, *A. baumannii*, *A. calcoaceticus*, and *St. aureus* [13]. Similarly, a study combining shotgun metagenome sequencing, machine learning, and culture-based methods aimed to investigate the gut microbiome of livestock, workers and their households, and microbial communities in carcasses and soil. The results revealed the most abundant microbial community composed of *E. coli*, *Lactobacillus johnsonii*, and *Lactobacillus salivarius* in chicken samples. Furthermore, opportunistic pathogens were also found, including 4 ESKAPE pathogens, *Ent. faecium*, *K. pneumoniae*, *P. aeruginosa*, *Enterobacter* species, and *E. coli* in broiler chicken feces, broiler chicken carcasses, and human samples [14]. The study by Gao et al. investigated the gut microbiome and resistome of residents in a swine farming village and their environmental relevance through a metagenomic sequencing approach. The findings of this study highlight the presence of *Enterobacteriaceae*, *Moraxellaceae*, *Burkholderiaceae*, *Bacteroidaceae*, and *Streptococcaceae families*, and, at the species level, *K. pneumoniae*, *Shigella flexneri*, and *A. baumannii* [15].

It is interesting to note the persistence of certain bacterial families in the studies mentioned and in our findings, suggesting a high prevalence and recurrence of *Enterobacteriaceae*, *Moraxellaceae*, and *Pseudomonadaceae.* This prevalence can be attributed to the ubiquity of these bacterial families in the gastrointestinal tracts of livestock animals and the environmental persistence of these bacterial families. *Enterobacteriaceae* can persist in soil, water, and manure, facilitating their spread into others farm environments. While *Pseudomonadaceae* can form biofilms, enhancing their survival [14,16,17]. This scenario can be further aggravated by the bacterial ability to acquire and spread AMR with well documented horizontal gene transfer by the *Enterobacteriaceae* family and intrinsic resistance mechanisms by *Pseudomonadaceae*, especially *P. aeruginosa*. Moreover, the use of antibiotics in livestock farming creates selective pressure favoring these resistant strains [14,17,18].

To the best of our knowledge, our work is the first to characterize plasmid replicons from diverse samples and locations using a resistome approach. We observed the dominance of the *Enterobacteriaceae* family with 37 different replicons found. This finding is consistent with previous studies showing *Enterobacteriaceae* as major carriers of plasmid-mediated resistance [19]. Moreover, another study used genomic epidemiology to assess any influence of livestock species and farm antimicrobial usage on antimicrobial resistance genes present in Enterobacterales collected from food animals. The results revealed 522 plasmids found on different livestock farms, including pEC4115, p011, IncY, IncX4, IncX1, IncR, IncN, Incl2, Incl1, Incl-G, IncHI2, IncFII, IncFIC, IncFIB, IncFIA, Col-like, IncHI1A, and IncBOKZ [20].

Notably, *E. coli*, *St. aureus*, *K. pneumoniae*, and *S. enterica* harbored the highest number of different replicons, with 66, 54, 50, and 37 plasmid replicons, respectively, suggesting that they are particular prone to acquiring and maintaining diverse plasmids, potentially contributing to their role as important pathogens and reservoirs of antibiotic resistance [19,21]. Finally, it is possible to observe the high frequency of Col, IncF, and IncH plasmids across multiple species, which aligns with previous research that showed these replicon types often associated with multidrug resistance and having a broad host range [22].

### 3.2. Antimicrobial Resistance Genes

Bacterial communities can drive and shape ARG prevalence [13]. As evidenced by the inoculum effect, cell density can increase or decrease AMR through drug enzymatic degradation, pH modulation, and growth bistability driven by the heat-shock response [23,24,25,26,27]. Furthermore, antibiotic-resistant cells can also reduce or accentuate the antibiotic effects close to susceptible cells [28,29,30]. Interestingly, there is a trade-off between resistant cells and susceptible cells. While the former group can increase the antibiotic threshold for the spread of plasmids carrying resistance genes to susceptible cells, the latter group is able to indirectly slow the growth of resistant populations [31].

Congruent with our data, a study, using a resistome approach, explored the seasonal dissemination of ARGs in a livestock farm and found *Pseudomonas* as a potential ARG pathogenic host, specifically *blaTEM-1*, in feces. Furthermore, *Pseudomonas* and *Acinetobacter* were identified as potential pathogenic hosts of multiple ARGs in soil [17]. Similarly, the work developed by Pitta et al. compared the microbiome and resistome distribution across different farm sectors. Among the aminoglycoside-N-acetyltransferases, the *aac3* ARG gene was the most abundant in dairy cow feces and was carried by the *Enterobacteriaceae* family. The *ant(3′)* were distributed in manure and lagoon samples and were mostly carried by Proteobacteria, while *ant6* was carried by Clostridia, Bacilli, *Actinomycetaceae*, and Bacteroidetes. The *ant9*, *blaCFX*, *blaACI*, and *blaCT* were carried by Bacteroides, Firmicutes, and Proteobacteria members, respectively [32]. The new data presented in our study, along with supporting data from the literature, reaffirms the high abundance of ARGs in livestock, which can vary from 10^6^ to 10^11^ copies/g dry weight or 10^6^ to 10^12^ copies/mL for absolute abundance, and 10^−3^ to 10^−1^ ARG copies/16S ribosomal RNA [33].

Munk et al. analyzed the resistome of pig and poultry livestock in nine European countries, quantifying 407 AMR genes [34]. In Spain and several other European countries (Belgium, Bulgaria, Germany, Denmark, France, Italy, the Netherlands, and Poland), the most prevalent antibiotic resistances in pig and poultry samples were to tetracyclines, macrolides, and aminoglycosides, with some β-lactam resistance [34]. Xiao et al. found fluoroquinolone resistance genes exclusively in Chinese samples, with no detection in Dutch or French swine fecal samples [35]. By contrast, Munk’s study identified fluoroquinolone resistance in poultry samples from various European countries [34]. Notably, Joyce et al. were pioneers in detecting fluoroquinolone resistance genes (*qnrB* and *qep*) in pig fecal metagenomes [36]. In our study, it was possible to identify quinolone resistance genes present also in 10 bacterial families of 38 total bacterial families carrying ARGs. We also identified aminoglycosides, beta-lactams, tetracyclines, and cephalosporins as the most prevalent ARGs, followed by macrolides. This variation can be attributed to the diverse livestock sources included in our study. Additionally, the results show certain correlation among veterinary antimicrobial usage data from the European Medicines Agency’s ESVAC with AMR gene prevalence. This analysis revealed a strong correlation between countries with similar antimicrobial consumption patterns, suggesting a direct link between antibiotic use and the development of resistance, considering country-specific usage of an antimicrobial drug [34].

Notably, Joyce et al. analyzed the fecal resistome of swine, identifying 257 total ARGs, which comprised a core resistome (56 ARGs), including five genes conferring resistance against tetracycline (*tetW*, *tetQ*, *tet44*, *tet37*, *tet40*), along with an additional 201 ARGs constituting the accessory resistome [36]. These findings were obtained from swine raised without antimicrobials, highlighting that, while antimicrobial use (AMU) is a significant factor, there are other influences affecting the acquisition and selective pressure of ARGs in swine. In a comparative analysis, Xiao et al. identified ARGs in swine samples that conferred resistance to bacitracin, cephalosporins, macrolides, streptogramin B, and tetracyclines [35]. Our data, however, reveals a different pattern of prevalence. Tetracycline resistance genes, particularly *tet(A)*, *tet(B)*, *tet(M)*, and *tet(O)*, emerged as the most prevalent in our samples. Beta-lactam and macrolide resistance genes were present but less common.

The higher prevalence of tetracycline resistance genes compared to those conferring resistance to aminoglycosides or beta-lactams in our study may be attributed to geographical variations in antibiotic usage patterns. This discrepancy highlights the importance of considering regional differences when analyzing antibiotic resistance profiles across different populations and environments. The samples in the study by Xiao et al. were obtained from China, Denmark, and the United States, each with distinct antibiotic usage practices. In China, antibiotics were used for growth promotion, whereas in Europe, this practice has been banned since 2006. These varying approaches to antibiotic use in livestock likely contribute to the differences observed in ARG prevalence between studies, underscoring the complex relationship between antibiotic usage policies and the development of resistance in bacterial populations [35].

Munk et al. identified a core resistome with 33 ARGs [34], while Xiao et al. found eight ARGs, including *cat(pC194)*, *ermB*, *ermF*, *inuA*, *nimJ*, *optrA*, *tet(40)*, and *aac(6′)-Im* [34]. Although our data has not delimited core resistome, some of those genes were prevalent in our samples, such as *ermB*, *aac(6′)*, a variation of *aac(6′)-Im*, *tet(40)*, and *cat(pC194*). Those previous studies did not identify colistin resistance *mcr-1*, *mcr* variant genes, or the *blaNDM* genes, while our data obtained several types of *bla*, including *blaNDM* and eight variations of *mcr-1* [34,35,36].

### 3.3. Bla Genes

Yang et al. analyzed swine and cattle waste, identifying *blaCMY-2*, *blaSHV*, *blaDHA*, and *blaKPC-2* and *blaCMY-2*, *blaSHV*, *blaSPM-1*, and *blaVIM-2*, respectively. Our study aligns with previous research on the prevalence of ESBL genes in livestock waste. Yang et al. report lower detection rates for genes like *blaNDM* and *blaVIM* in farm samples, while our data shows a higher prevalence of these carbapenemase genes. This discrepancy could be attributed to differences in antibiotic use practices, geographical variations, or the specific bacterial populations studied. Our study detected a wider array of β-lactamase genes, including uncommon types, such as *blaGOB*, *blaLUT*, and *blaMUS*, likely stemming from a more diverse sampling approach [37]. Kim et al. identified beta-lactamases in livestock samples (chicken, swine, and cattle), where *ampC*, *blaTEM*, *blaOXA*, *blaCTX-M*, *cfxA*, *cepA*, and *qnrS* were specifically found in chickens. The sampling strategy may have enhanced the identification of rare resistance genes, including more diverse animal species and a longer period of study, offering a more comprehensive picture of the β-lactamase landscape [38]. The higher abundance of *blaCTX-M* cluster 1 genes found by Munk in Spanish poultry herds, along with those in Italy, Poland, and Belgium, may be linked to the higher use of fluoroquinolones in these countries, as *qnr* and *bla_CTX-M_* genes are often co-located on ESBL plasmids. However, the relationship between veterinary cephalosporin usage and *bla_CTX-M_* levels remains unclear, highlighting the complex nature of ARG dissemination [34].

### 3.4. Linking Antimicrobial Resistance in Livestock to Clinical Cases

There is a clear connection and interdependency among human health, animals, and the environment, which is the dogma behind the One Health approach, that aims for the prevention, rapid response, and management of infectious diseases, antimicrobial resistance, and food safety at the local, regional, national, and global levels using these tripartite factors [39]. Since human health, animals, and the environment are linked, connections and relationships are expected to be found among them. Maciel-Guerra aet al. conducted a longitudinal study on a poultry farm and a connected slaughterhouse and found that 14% of species and 76% of ARGs found in humans were also present in the chickens [14]. Moreover, 11 clinically relevant ARGs were present in the same mobile ARG patterns in both hosts. This study also found 6 chicken metagenome-assembled genomes of the 566 constructed that clustered at a 99% average nucleotide identity with human samples.

By using whole-genome phylogenetic analysis and network analysis based on single nucleotide polymorphisms (SNPs), the work developed by Peng et al. found highly interrelated non-pathogenic and pathogenic *E. coli* strains with phylogenetic intermixing, and a high prevalence of shared multidrug resistance profiles amongst livestock, human, and the environment characterized by a high prevalence of *E. coli* isolates that are resistant to multiple antimicrobials [40]. The most prevalent AMR profile identified in the cohort included resistance to penicillins, aminoglycosides, mono beta-lactams, cephalosporins, chloramphenicol, quinolones, tetracyclines, sulfonamides, and polymyxins. This AMR profile was observed across different sequence type clusters. Notably, three of these clusters contained both chicken and human isolates, while one cluster contained human and environmental isolates. This indicates a significant overlap in AMR profiles between the different hosts and the environment within the livestock farming context studied [40]. Our data also encompasses aminoglycosides, tetracyclines, and cephalosporin resistance genes, which can be further explored to establish share profiles with humans’ resistances.

Sun et al. demonstrated a correlation among human gut microbiota and resistome and exposure to the high-risk swine farm environment through taxonomic and functional remodeling. The gut microbiota and resistome of individuals exposed to swine farms showed enrichment in potentially pathogenic taxa and antimicrobial resistance genes, such as *mcr-1*, *blaTEM* and *blaCTX-M*, *qnrS*, *tet(X)*, *mcr-1*, *fosA3*, *mph(B)*, and *arr-3* [41]. Similarly, our study also identified the *blaTEM*, *blaOXA*, *blaNDM*, and *qnrS* genes, reflecting a high prevalence of resistance to beta-lactams, aminoglycosides, tetracyclines, and macrolides across both environments. Furthermore, comparing the results obtained by Sun et al. with ours, it is possible to note that both studies report aminoglycosides, beta-lactams, and tetracyclines as more abundant resistance classes. The swine farm study found high levels of beta-lactamases and aminoglycoside resistance, while our study also reports aminoglycosides (21.36%), beta-lactams (17.72%), and tetracycline (11.75%) as the top resistance categories.

Interestingly, Hu et al. compared the gut metagenome of chicken samples from suburban farms in Beijing, China, to reference gut microbiome genes from the Chinese Gene Catalog. The results revealed that about 35% of human- and animal-associated strains carried mobile ARGs. Moreover, human gut microbiomes shared higher mobile ARGs with chicken gut microbiomes (36 genes) and the fewest with cattle (10 genes). The network analysis of ARGs from Europe, China, and America showed that the European and Chinese catalogs shared higher mobile ARGs (69 genes). However, all three gene catalogs shared more ARGs with chickens than other animals. The authors also identified 84 mobile ARGs shared between at least two gut datasets, where 41 were recently transferred between human and animal guts, covering six major antibiotic classes, including tetracyclines (11 genes), aminoglycosides (10 genes), macrolide–lincosamide–streptogramin B (MLSB) (9 genes), chloramphenicols (5 genes), beta-lactams (3 genes), and sulfonamides (3 genes) [42].

Further analysis of 2613 genomes of human-associated pathogens revealed that 33 of these 41 mobile ARGs were present in 47 species and 403 genomes, often in human pathogens. The *tet(M)* gene was the most widespread, found in 13 species, especially in *Streptococcus agalactiae* (95 genomes). *Str. agalactiae*, *E. coli*, and *Str. suis* were the three species carrying the most ARGs shared between human and animal guts, each harboring seven genes. Furthermore, the *erm(F)* gene was found in *Bibersteinia trehalosi* and associated with known mobile genetic elements. Similarly, the *ant(6)-Ia* gene, shared between pig and human guts, also demonstrated the role of mobile genetic elements in ARG mobility [42]. Comparing our data with this study, both highlight the high distribution of *E. coli*, *S. enterica*, *K. pneumoniae*, and *St. aureus*. There are also similarities in the resistance patterns to the antibiotic classes. While we found resistance coding genes to aminoglycosides, beta-lactams, tetracyclines cephalosporins, and macrolides, Hu et al. found resistance to tetracyclines, aminoglycosides, MLSBs, beta-lactams, and sulfonamides as the most prevalent resistance classes. It is important to emphasize the role of the mobile genetic elements (such as those associated to integrons), since both works found these elements, with specific examples like the *erm(F)* and *ant(6)-Ia* genes. These comparations of resistance to antibiotic classes and mobile genetic element presence underscore the widespread occurrence of ARGs across human, animal, and environmental ecosystems and reinforces the importance of the One Health approach to deal with the global antibiotic resistance threats.

Another study characterized the resistome and bacterial microbiome of farm chickens, live poultry markets, workers, and control individuals [43]. The *mcr-1* and *tet(X3)* genes were identified in chickens, live poultry markets environments, and live poultry market workers. Moreover, the same genes were also found in human pathogens, indicating potential cross-domain transfer between animals and humans via the environment [43]. As in our study, they also identify pathogenic bacteria such as *Enterobacteriaceae.* In their case, this bacterium was found in both human and animal samples, indicating a shared reservoir, which can also suggest a specific bacterial family’s dominance in livestock and human environments.

Kim et al. analyzed healthy individuals and those with *C. difficile* infection (CDI) targeting the resistome, and found a difference between the ARGs obtained. CDI patients showed the highest prevalence, the ARGs being more prevalent to aminoglycosides, β-lactams, quinolones, and MLS (macrolides, lincosamides, streptogramins). Interestingly, our data also showed a higher presence of ARGs to aminoglycosides, as well as several variations of *bla* genes and ARGs to MLS and quinolones. The *bla* genes observed in the Kim et al. study were *bla_TEM_*, *bla_OXA_*, *bla_SHV_*, and *bla_CTX_*_-*M*_. By comparison, our data revealed a prevalence of 16.98% for *bla_TEM_*, 13.84% for *bla_OXA_*, and 5.03% for *bla_CTX_*_-*M*_. Notably, these *bla* genes were present in both healthy individuals and especially in CDI patients in the Kim et al. study, with similar proportions observed in our dataset. The presence of these genes across different patient groups underscores their widespread distribution and potential clinical significance [44]. They also noted high prevalence of *qnrS* genes in CDI patients, where 61.5% of all patients had at least one *qnr* resistance genes (46.15%). By contrast, our data showed 0.55% of *qnr* genes, including *qnrB*, *qnrD*, *qnrS*, *qnrVC*, and *qnrA* [45]. Pustam et al. also identified *qnrB* in *K. pneumoniae* isolates from hospitals in Trinidad, West Indies. These encoded genes do not replace antibiotic active sites; instead, they provide protection to the enzymes DNA gyrase and topoisomerase IV [45]. Filippa et al. also detected 66.67% of *qnrB*, *bla_CTX-M15_*, and *aac(6′)-Ib-cr* isolated from patients in France, specifically in *K. pneumoniae* [46]. We also found these genes in *E. coli*, *K. pneumoniae*, *Morganella morganii*, *Ent. cloacae*, and *Aeromonas caviae*. The presence of these resistance genes in clinical samples indicates a widespread distribution of antibiotic resistance among diverse bacterial groups, correlating with pathogens found in livestock.

Pustam et al. also revealed the presence of non-ESBL SHV variants from patients, specifically *bla_SHV-1_* and *bla_SHV-11_* [45]. These enzymes are capable of hydrolyzing narrow-spectrum β-lactams, including cefamandole and cefoperazone, and confer resistance to penicillins. The presence of these non-ESBL variants is particularly noteworthy in *K. pneumoniae* isolates, where they contribute to the species’ intrinsic resistance to ampicillin and other early-generation β-lactams [45].

Rahmat et al. identified clinical *K. pneumoniae* isolates with at least two β-lactamase encoding genes [47]. Our data present different β-lactamase genes, but in the same bacteria. Considering our spectrum of different samples, we also identified the presence of *bla_NDM_*, *bla_VIM_*, *bla_OXA_*, *bla_KPC-3_*, *bla_CMY-2_*, and *bla_IMP_.* The plasmid mediated-quinolone resistance genes found matched with our findings, being *OqxA*, *OqxB*, *qnrB1*, *qnrB19*, *qnrB2*, *qnrB4*, *qnrB91*, *qnrD1*, and *qnrS1*. The prevalence of ESBL genes, such as *bla_CTX-M-15_*, *bla_TEM_* variants, *bla_OXA_* types, and *bla_SHV_*, in our isolates correlates to the findings from previous studies [45,47]. This similarity suggests that AMR is mediated by both intrinsic and acquired genetic elements, which may be located on chromosomes or mobile genetic structures.

Our results reveal a widespread distribution of these ARGs across various bacterial species, particularly within the *Enterobacteriaceae* family. Notably, we observed *bla_CTX-M-15_* in several species including *K. pneumoniae*, *E. coli*, and *E. cloacae*. The *bla_TEM_* variants (such as *bla_TEM-1A_*, *bla_TEM-1B_*, and *bla_TEM-1C_*) were detected in multiple bacterial species, including *K. pneumoniae*, *E. coli*, *and A. baumannii.* The presence of *bla_OXA_* variants (e.g., *bla_OXA-1_* and *bla_OXA-48_*) in *K. pneumoniae* and *E. coli*, along with *bla_SHV_* variants in *K. pneumoniae*, further underscores the widespread nature of these resistance determinants. Interestingly, we also found the *aac(6′)-Ib-cr* gene co-occurring with ESBL genes in several isolates, particularly in *K. pneumoniae* and *E. coli*. This co-occurrence suggests the potential involvement of specific plasmids in facilitating the dissemination of these resistance genes through vertical transfer mechanisms or even bacteriophages by lysogeny [48,49].

## 4. Materials and Methods

### 4.1. Samples

Bacterial isolates were collected from different farms and facilitated by research and official animal health laboratories in Spain. Samples were obtained from multiple types of animal species and food and food-related environments. In detail, the samples were collected over a period of 10 years, between 2015 and 2024, with a national distribution, including all regions of Spain. The nature of the samples varied, being mainly isolated from animals (88%), and from the food environment (food and food facilities) (12%). The types of samples of animal origin were mainly isolated from fecal content, but also from exudates and clinical samples. The main animal species from which the isolates were obtained were as follows: pig (69%), sheep (14%), cattle (11%), goats (4%), and other species (i.e., fish farm animals) (1%). All samples of food origin were associated with foods of animal origin. Specifically, the majority of isolates were obtained from milk and dairy products (72%) and meat and meat products (28%), as well as from factories where these foods were elaborated. Bacterial taxonomic confirmation was performed using classical microbiological techniques supported by biochemical identification using biochemical procedures and MALDI-TOF mass spectrometry. After confirmation of strain purity, DNA was purified from axenic cultures using the Qiagen DNA blood & tissue kit (QIAGEN, Hilden, Germany), following the manufacturer’s instructions. Quantification of the DNA concentration and further DNA from libraries hereafter was performed using a Qubit^®^ fluorometer (Invitrogen, Waltham, MA, USA).

### 4.2. Sequencing

WGS libraries were prepared from 1 ng of bacterial DNA by using the Nextera XT DNA Library Preparation Kit (Nextera, Juno Beach, FL, USA) as previously described [50]. *Clostridia* and *S. aureus* libraries were prepared using Illumina DNA Prep following the manufacturer’s instructions. The concentrations of each library were adjusted to 4 nM to obtain equimolar DNA concentrations in a single pool of libraries and sequenced in a MiSeq platform using the 2 × 300 cycle V3 Kit (Illumina, Hayward, CA, USA) to obtain at least 30× coverage of the draft genome.

### 4.3. Accession Numbers

The genomes of all bacterial isolates have been deposited within NCBI/GenBank under the number SUB14790332.

### 4.4. Genomic Analysis

We used a bioinformatic tool previously successfully validated for bacterial whole genome analysis [50]. It is a pipeline for conducting bacterial WGS analysis directly from raw sequences from Illumina sequencing platforms. Adaptors in the raw reads and sequences that do not meet the length and quality criteria were filtered using Trimmomatic and PRINSEQ [51,52]. In addition, Kraken 2 software was used to classify sequences taxonomically, as an additional quality control [53]. Reads that passed the quality control were then assembled into a draft genome with SPAdes v3.15.2 [54]. The quality of the assemblies was evaluated by using QUAST 5.0.2 [55]. Draft genomes were then annotated using Prokka v1.14.6 [8] and the resulting annotation files were used for a pangenome comparison based on the presence or absence of predicted genes between samples by using Roary 3.11.2 [56]. Multilocus sequence typing (MLST) analysis was performed by using the MLST software 2.23.0 and the PubMLST database (https://pubmlst.org, accessed on 3 December 2024). Screening of the draft genome was performed by using BLASTn 2.10.1+ and ABRicate 1.0.1 [57] against ResFinder 4.6.0 [58] and CARD [59] databases for the analysis of ARGs, and against the PlasmidFinder 2 database [60] and the Virulence Factors Database (VFDB) [61] for the analysis of plasmid replicons and virulence genes, respectively.

### 4.5. Metadata Analysis

The R studio (version 2023.06.1+524) (http://www.rstudio.com/, accessed on 3 December 2024) was used to carry out different heatmaps using the ggplot2 package (https://ggplot2.tidyverse.org, accessed on 3 December 2024).

## 5. Conclusions

Our study represents an initial step to characterize the resistome in primary production in Spain. A large number of bacterial isolates (6222) were sequenced and AMR information was obtained. We identified a high number of ARGs (1072) coding for a wide type of AMR, potentially conferring resistance to 81 antimicrobial drugs. In addition, our study highlights that the presence of ARGs in the livestock in Spain is mainly associated to those specific bacteria with a global burden of antibiotic resistance, including *E. coli*, *Salmonella* spp., *K. pneumoniae*, *St. aureus*, and *A. baumannii*. A relevant finding of our study was the high number of MDR bacterial isolates (2841; 45.67%) identified, which was significantly higher than in previous studies conducted (but those with a notably more limited number of bacterial isolates studied). Additionally, a notably high number of mobile genetic elements were also identified; 79 different plasmid types were detected, highlighting the potential for horizontal gene transfer. In summary, our study represents a comprehensive characterization of AMR genes circulating in bacteria from primary production providing initial crucial insights into the ecology of AMR in Spanish livestock.

## Figures and Tables

**Table 1:** Bacterial taxa without AMR genes identified in this study.

Bacterial Taxa	Number of Isolates
Family	
Acetobacteraceae	1
Brucellaceae	201
Burkholderiaceae	100
Caulobacteraceae	1
Francisellaceae	223
Leuconostocaceae	2
Mycoplasmataceae	12
Nocardiaceae	1
Paenibacillaceae	6
Peptostreptococcaceae	126
**Species**	
*Acetobacter ghanensis*	1
*Aerococcus sanguinicola*	1
*Aerococcus urinae*	1
*Aerococcus urinaeequi*	3
*Aerococcus urinaehominis*	1
*Aerococcus viridans*	1
*Aeromonas veronii*	1
*Anoxybacillus flavithermus*	4
*Bacillus amyloliquefaciens*	1
*Bacillus atrophaeus*	1
*Bacillus cereus*	50
*Bacillus megaterium*	1
*Bacillus velezensis*	1
*Lysinibacillus* sp.	1
*Brucella abortus*	129
*Brucella ceti*	17
*Brucella melitensis*	35
*Brucella pinnipedialis*	2
*Brucella* sp.	1
*Brucella suis*	17
*Burkholderia contaminans*	12
*Burkholderia multivorans*	37
*Burkholderia vietnamiensis*	2
*Cupriavidus metallidurans*	2
*Ralstonia mannitolilytica*	27
*Ralstonia pickettii*	20
*Brevundimonas diminuta*	1
*Clostridium septicum*	8
*Clostridium sphenoides*	1
*Clostridium tetani*	3
*Delftia tsuruhatensis*	1
*Corynebacterium striatum*	1
*Corynebacterium ulcerans*	4
*Corynebacterium variabile*	1
*Atlantibacter hermannii*	2
*Pantoea alhagi*	3
*Francisella tularensis*	223
*Lactobacillus curvatus*	2
*Lactobacillus mali*	4
*Lactobacillus plantarum*	1
*Acinetobacter lwoffii*	1
*Providencia alcalifaciens*	2
*Mycoplasma orale*	12
*Rhodococcus aetherivorans*	1
*Brevibacillus laterosporus*	2
*Paenibacillus cellulositrophicus*	1
*Paenibacillus polymyxa*	2
*Paenibacillus xylanexedens*	1
*Pasteurella multocida*	7
*Paeniclostridium sordellii*	5
*Pseudomonas fluorescens*	1
*Pseudomonas koreensis*	1
*Pseudomonas protegens*	3
*Rahnella aquatilis*	1
*Serratia fonticola*	1
*Serratia proteamaculans*	1

**Table 2:** Bacterial MDR (coding genes) families and species identified.

Family	Species	Number of Resistance Genes	% Strains
Enterobacteriaceae	*Escherichia coli*	1680	32.93%
	*Klebsiella pneumoniae*	390	6.30%
	*Salmonella enterica*	281	9.23%
	*Citrobacter freundii*	21	0.40%
	*Enterobacter cloacae*	25	0.40%
	*Enterobacter hormaechei*	38	0.74%
Pseudomonadaceae	*Pseudomonas aeruginosa*	401	6.44%
	*Pseudomonas putida*	30	0.56%
Moraxellaceae	*Acinetobacter baumannii*	54	0.87%
Staphylococcaceae	*Staphylococcus aureus*	296	5.79%
Clostridiaceae	*Clostridium perfringens*	209	4.60%
Streptococcaceae	*Streptococcus suis*	40	0.84%
	*Lactococcus garvieae*	150	3.57%
Enterococcaceae	*Enterococcus faecium*	50	0.80%
Campylobacteraceae	*Campylobacter coli*	70	1.74%
	*Campylobacter jejuni*	29	0.69%
Peptostreptococcaceae	*Clostridioides difficile*	47	0.76%
Yersiniaceae	*Yersinia enterocolitica*	28	0.67%
Morganellaceae	*Morganella morganii*	10	0.16%
	*Providencia rettgeri*	6	0.14%

## Data Availability

Information and data can be facilitated upon request.
